# Study on the effectiveness and safety of ciprofol in anesthesia in gynecological day surgery: a randomized double-blind controlled study

**DOI:** 10.1186/s12871-023-02051-x

**Published:** 2023-03-25

**Authors:** Yan Man, Hongyi Xiao, Teng Zhu, Fanceng Ji

**Affiliations:** 1 School of Anesthesiology, Weifang Medical University, Weifang, 261053 China; 2 Department of Anesthesiology, Weifang People’s Hospital, Weifang, 261041 China

**Keywords:** Ciprofol, Propofol, Day surgery

## Abstract

**Backgroud:**

ciprofol is a new type of intravenous anesthetic, which is a tautomer of propofol, with the characteristics of less injection pain, less respiratory depression and higher potency, but little clinical experience. The aim of this study was to observe the efficacy and safety of the application of ciprofol in ambulatory surgery anesthesia in gynecology.

**Methods:**

128 patients were selected to undergo gynecological day surgery under general anesthesia, and the patients were randomly divided into the ciprofol group and the propofol group, with 64 cases in each group. During anesthesia induction, the ciprofol group was infused at a time limit of 0.5 mg/kg for one minute, and the propofol group was infused at a time limit of 2 mg/kg for 1 min. The overall incidence of adverse events was the primary outcome for this study, while secondary outcomes included the success rate of anesthesia induction, the time of loss of consciousness, the time of awakening,top-up dose and frequency of use of rescue drugs.

**Results:**

The overall incidence of adverse events was significantly lower in the ciprofol group compared with the propofol group (56.2% vs. 92.2%,P < 0.05). The success rate of anesthesia induction of ciprofol and propofol group was 100.0%. The time of loss of consciousness of the ciprofol group was longer than that of the propofol group (1.6 ± 0.4 min vs. 1.4 ± 0.2 min, P < 0.05). The time of awakening was not statistically significant (5.4 ± 2.8 min vs. 4.6 ± 1.6 min, P > 0.05). The number of drug additions and resuscitation drugs used were not statistically significant.

**Conclusions:**

Compared with propofol, ciprofol had a similar anesthetic effect in gynecological ambulatory surgery, and the incidence of adverse events in the ciprofol group was lower.

## Introduction

Day surgery has become popular in different countries because of its advantages in reducing hospitalization time, improving bed utilization and reducing hospitalization costs. With the development of medical technology, the proportion of day surgery is growing rapidly. Guidelines from the Association of Anaesthetists and the British Association of day surgery[1] argue that day surgery anesthesia should be selected with the goal of minimizing patient stress and optimizing comfort. Therefore, general anesthesia has become the most commonly used anesthesia method for ambulatory surgery. Day surgery requires anesthetic drugs with rapid onset, rapid elimination, short duration of action, good sedative and analgesic effects, minimal effects on cardiopulmonary function, and no serious adverse effects or discomfort[2]. Propofol is a widely used intravenous anesthetic with the advantages of rapid onset, rapid recovery and no accumulation.Due to its disadvantages such as dose-dependent blood pressure reduction and injection pain, its application in the elderly, circulatory dysfunction, etc. is limited[3, 4].Injection pain is one of the most common adverse events of propofol, the incidence of Injection pain in adults is 28-90%[4], Ciprofol is a new type of intravenous anesthetic developed independently in China, which is a short-acting γ-aminobutyric acid A receptor (γ-aminobutyric acid subtype A receptor (GABAA) agonist,and has now completed a Phase 3 clinical trial[5, 6]. In the previous experiment [7], it was proved that ciprofol has the characteristics of fast onset of action, rapid recovery, no accumulation, less pain and small respiratory depression after injection, which has potential clinical application value. However, there is still little experience in the application of ciprofol in clinical practice,,More trials are needed to analyze the safety and efficacy of ciprofol. This study intend to use a randomized double-blind control to explore the safety and efficacy of ciprofol by comparing the adverse events and anesthesia effects between ciprofol and propofol in gynecological day surgery, in order to provide reference for clinical application.

## Materials and methods

### Patients and study protocol

The study was conducted at Weifang People’s Hospital, has been approved by the hospital ethics committee(2,021,037). This study was registered with the Chinese Clinical Trial Registry (ChiCTR2100053444) in 21/11/2021 and informed consent was signed by patients or their legal guardian.

This study was a randomized double-blind controlled study,and the primary endpoint was the overall incidence of adverse events.Patients (18 ~ 64years),with American Society of Anesthesiologists physical classification status I or II, BMI between 18 and 28 kg/m^2^_,_ who were about to undergo gynaecological ambulatory surgery from January 2022 to June 2022 were eligible.Patients were excluded if they sufered from egg/soy/propofol allergies,significant cardiovascular, respiratory or hepatic and renal diseases in this study.In addition, women who were pregnant, or planning to become pregnant were excluded.An independent investigator used random number table to assign patients to the ciprofol group and the propofol group, with 64 patients in each group.The distribution list is placed in an envelope that is opened by the nurse anesthetist on the day of surgery to prepare study medications in the anesthesia preparation room. The nurse anesthetist is not directly involved in patient care. Anesthesiologist performed anesthesia without knowing the grouping. Participants and outcome assessors were blinded to group allocation.

The patient had no pre-anesthetic medication.Following arrival in the operating room, patients were monitored via electrocardiography, pulse oximetry, bispectral index(BIS,Mindray), and continuous noninvasive blood pressure and established intravenous access of the upper limb.Intravenous flurbiprofen axetil (50 mg), dexamethasone (5 mg) were used to start general anesthesia induction, followed by pump injection with Medical Syringe Pump(Silugao) for 60 s of ciprofol (0.5 mg/kg; Liaoning Haisco Pharmaceutical Co.Ltd,National Drug Administration (NDA)H20200013)or propofol (2 mg/kg; Fresenius Kabi AB,National Drug Administration (NDA)HJ20170305). The time of loss of consciousness from the beginning of study drug administration were assessed every 5 s by calling for eye opening or by mild prodding or shaking.Once the patient reached the modified observer’s assessment of alertness/ sedation (MOAA/S) ≤ 1 (no response after mild prodding or shaking), 0.2 mg/kg mivacurium chloride and 20ug/kg alfentanil were administered immediately. After the spontaneous breathing disappears, oxygen was administered under face mask pressure, and after the skeletal muscles were relaxed, the glottis was exposed with a visual laryngoscope and 2% lidocaine(3 ml) was sprayed into the subglottis using Single-use ENT anesthetic nebulizer.Then a tracheal catheter was inserted and properly fixed. Mechanical ventilation was performed with parameters set at 6 ~ 8 ml / kg for V_T_,60% for FiO2, 12 ~ 16 times / min for RR, 1: 2 for I:E. If the patient failed to achieve MOAA/S ≤ 1 within 1 min after the full induction dose was administered, one-half of the initial dose was given.If the patient failed to achieve MOAA/S ≤ 1 was not reached within 2 min, it would be regarded as a failure of general anesthesia induction of the drug in this study.Anesthesia was maintained with ciprofol (1 mg/kg/h) or propofol(5 mg/kg/h) and alfentanil (40 ug/kg/h). During the operation, when blood pressure or heart rate rose to 20% of the basal value,study druy( 0.05 ml/kg) was given; when the blood pressure dropped by 30% of the basal value, ephedrine (6 mg) was given; when the heart rate was less than 50 beats/min, atropine(0.3 mg) is given. Stop the infusion of all medications when the surgical operation is stopped.After the endotracheal tube was removed, patient was transferred to the postanesthesia care unit (PACU).

**Primary outcomes**: The overall incidence of adverse events. Overall adverse event is defined as an event that occurs in the perioperative period that affects the safety of anesthesia.Overall adverse events included: (1) bradycardia (HR < 50 beats/min, > 30s); (2) Tachycardia (HR > 100 beats/min, > 30s) (3) Hypotension (30% reduction in SBP compared to baseline value); (4) Hypertension (SBP is 20% higher than baseline value); (5) injection pain(We asked patients if they feel pain in the arm when the drug was injected); (6) Intraoperative body movements(The patient had no conscious movement of the limbs).

#### Secondary outcomes included

(1) success rate of induction of anesthesia,(2) the time of loss of consciousness (time of initiation of study drug infusion to MOAA/S ≤ 1), (3) time of awakening (time of drug discontinuation to extubation), (4) study drug top-up doses, (5)rescue drug use.

## Sample size and statistical analysis

In type I error 0.05(bilateral), Power of test is 80%.The overall incidence of adverse events was approximately 36.4% in the ciprofol group, and 60.6% in the propofol group.Finally a total of 128 patients were includeda in this study.

Using SPSS 25. 0 Statistical software for data analysis. normal distribution measurement data is expressed as mean ± standard deviation (x ± s), with two independent samples t-test used for inter-group comparisons; The enumeration data is represented by the example (%) method, the inter-group comparison is represented by the χ^2^ test or the Fisher exact method. P<0. 05 indicates that the difference is statistically significant.

## Results

**Fig. 1:**
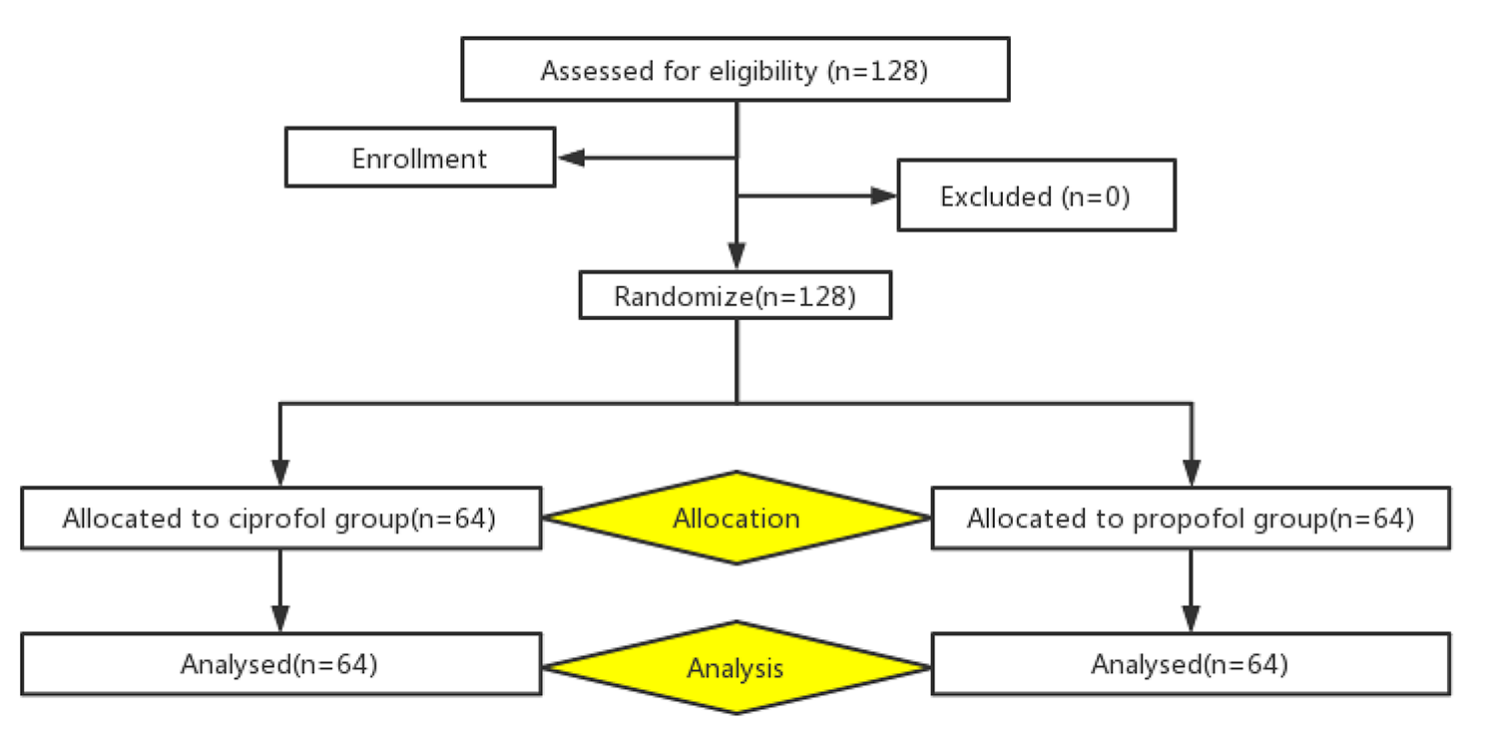
A fow chart of the current trial

128 patients were included in this study. Data from 128 patients were obtained for statistical analysis (Fig. 1). There were no significant differences between the ciprofol and propofol groups with respect to patient age, weight, height, BMI, ASA physical status, type of surgery and anesthesia time (Table 1).

**Table 1:** Comparison of the general situation of the two groups of patients

	Ciprofol group (n = 64)	Propofol group (n = 64)	P-value
Age,year,mean ± SDs	42.2 ± 9.46	44.1 ± 9.4	0.21
Weight,Kg,mean ± SDs	58.7 ± 6.1	59.8 ± 6.9	0.37
Height,cm,mean ± SDs	160.6 ± 4.4	160.2 ± 4.9	0.62
BMI,Kg/m^2^,mean ± SDs	22.8 ± 2.2	23.3 ± 2.6	0.21
ASA status,n(%)			
I	18(28.1)	14(21.9)	0.41
II	46(71.9)	50(78.1)	
Operation type,n(%)			
Uteroscope	48(75.0)	54(84.4)	
Conization of cervix	16(25.0)	9(14.1)	0.18
other	0(0.0)	1(1.6)	
Venipuncture site,n(%)			
Back of the hand	46(71.9)	33(51.6)	
Wrist	15(23.4)	22(34.4)	0.059
Forearm	0(0)	1(1.6)	
Elbow	3(4.7)	8(12.5)	
Induced dose,mg,mean ± SDs	29.4 ± 3.0	119.5 ± 13.9	
Maintenance Dose,mg,mean ± SDs	14.8 ± 9.8	85.6 ± 44.0	
Anesthesia time,min,mean ± SDs	19.4 ± 8.2	19.9 ± 6.5	0.68

A total of 137 adverse events occurred in 128 patients, of which 44 adverse events occurred in 64 patients in the ciprofol group and 93 adverse events occurred in 64 patients in the propofol group. Among those who had 2 or more adverse events in one person, 11% were in the ciprofol group and 48.5% were in the propofol group. The overall incidence of adverse events was significantly lower in the ciprofol group compared with the propofol group (56.2% vs. 92.2%,P < 0.05). The incidence of hypotension was the highest, accounting for 44.5% of the total adverse events. Those who required intraoperative ephedrine to boost blood pressure were 5 cases in the ciprofol group and 9 cases in the propofol group, of which 2 patients in the propofol group used it twice and the rest used used it once, with no statistically significant difference between the two groups (P > 0.05), as shown in Table 2.

**Table 2:** Incidence of intraoperative adverse events

	Ciprofol group	Propofol group	P-value
total adverse events, n(%)	44(56.3)	93(92.2)	0.000
Bradycardia,n(%)	7(10.9)	6(9.4)	0.770
Tachycardia, n(%)	1(1.6)	0(0.0)	1.000
Hypotension, n(%)	25(39.1)	36(56.3)	0.052
Hypertension, n(%)	4(6.3)	2(3.1)	0.437
Injection pain, n(%)	1(1.6)	49(76.6)	0.000
body movements,n(%)	6(9.4)	0(0.0)	0.037

The success rate of induction was 100% in both groups, and the comparison between the two groups was not statistically significant (P > 0.05).The time of loss of consciousness in the ciprofol group was longer than that in the propofol group (1.6 ± 0.4 min vs. 1.4 ± 0.2 min), which was statistically significant (P < 0.05). The time of awakening was not statistically significant in the two groups(P > 0.05).Top-up dose during the operation was not statistically significant in the two groups (P > 0.05), (Table 3).

**Table 3:** Secondary outcomes

	Ciprofol group	Propofol group	P-value
success rate of induction,n(%)	64(100)	64(100)	1.00
the time of loss of consciousness,min,mean ± SDs	1.6 ± 0.4	1.4 ± 0.2	0.00
the time of awakening,min,mean ± SDs	5.4 ± 2.8	4.6 ± 1.6	0.72
top-up dose,n(%)			
1	8(12.5)	9(14.1)	
2	4(6.3)	4(6.3)	1.00
3	0(0)	1(1.6)	
4	1(1.6)	0(0)	

**Figure 2:**
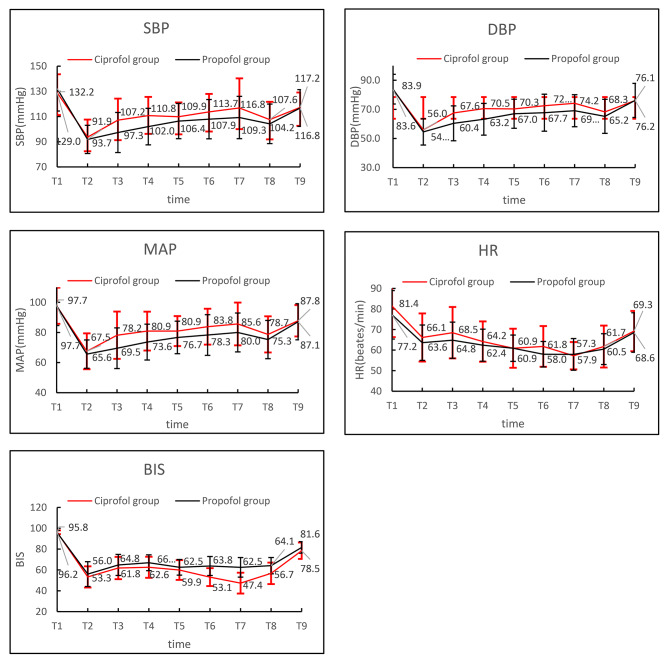
Changes in blood pressure, heart rate, and BIS at different time points during the patient’s perioperative period

The intraoperative blood pressure, heart rate, and BIS trends were similar, with a transient decrease in blood pressure after injection, followed by an increase in blood pressure and tending to stabilize. Compared with T1, SBP、HR were lower at T2 ~ T9 and the difference was statistically significant.The difference between SBP at T 1 and at T 3 ~ T 6 in the ciprofol group was lower than that in the propofol group(P < 0.05).The difference between BIS at T 1 and at T4、T 6 ~ T 8 in the ciprofol group was larger than that in the propofol group(P < 0.05)(Table 4、Fig. 2).

**Table 4:** Comparison of intraoperative hemodynamics and anesthesia depth

	T1	T2	T3	T4	T5	T6	T7	T8	T9
Ciprofol group									
SBP, mmHg,mean ± SDs	129.0 ± 14.6	93.7 ± 14.0*	107.2 ± 17.1*	110.8 ± 14.9*	109.9 ± 11.3*	113.7 ± 14.3*	116.8 ± 23.5*	107.6 ± 14.1*	117.2 ± 11.9*
HR, beats/min,mean ± SDs	81.4 ± 15.1	66.1 ± 11.7*	68.5 ± 12.5*	64.2 ± 9.8*	60.9 ± 9.5*	61.8 ± 9.9*	57.3 ± 6.7*	61.7 ± 10.2*	69.3 ± 9.8*
BIS, mean ± SDs	96.2 ± 1.8	53.3 ± 10.2*	61.8 ± 10.7*	62.6 ± 10.1*	59.9 ± 9.5*	53.1 ± 8.6*	47.4 ± 10.0*	56.7 ± 10.3*	78.5 ± 7.9*
Propofol group									
SBP, mmHg,mean ± SDs	132.2 ± 17.8	91.9 ± 11.2*	97.3 ± 15.9*	102.0 ± 14.5*	106.4 ± 14.0*	107.9 ± 15.6*	109.3 ± 16.8*	104.2 ± 15.7*	116.8 ± 14.6*
HR, beats/min,mean ± SDs	77.2 ± 11.8	63.6 ± 8.6*	64.8 ± 8.8*	62.4 ± 7.8*	60.9 ± 6.4*	58.0 ± 6.2*	57.9 ± 7.7*	60.5 ± 7.5*	68.6 ± 9.6*
BIS, mean ± SDs	95.8 ± 2.2	56.0 ± 11.9*	64.8 ± 10.1*	66.9 ± 7.6*	62.5 ± 7.5*	63.8 ± 9.1*	62.5 ± 9.4*	64.1 ± 7.8*	81.6 ± 5.6*
P-value									
SBP	0.269	0.423	0.001	0.001	0.119	0.166	0.381	0.198	0.848
HR	0.078	0.166	0.057	0.165	0.983	0.096	0.833	0.426	0.689
BIS	0.271	0.169	0.108	0.008	0.135	0.000	0.001	0.000	0.010

Note: T 1: Before Anesthesia, T 2: before endotracheal intubation, T 3:1 min after endotracheal intubation, T 4: at the beginning of surgery, T 5:5 min after surgery, T 6:10 min after surgery, T 7:15 min after surgery, T 8: At the end of Surgery, T 9: after extubation.Comparison with T1, *P < 0. 05

Figure 2 Changes in blood pressure, heart rate, and BIS at different time points during the patient’s perioperative period.

## Discussion

Ciprofol is a new type of intravenous anesthetic independently developed in China, its chemical name is 2-[(1R)-1-cyclopropylethyl]-6-isopropylphenol, which is an isomer of propofol, and the ciprofol group is introduced into the chemical structure of propofol, forming a chiral structure and increasing the stereo effect. By enhancing GABAA receptor-mediated ion channels, it influxes chloride ions, causing hyperpolarization of nerve cell membranes to achieve central nervous system suppression [8]. Because of its low injection pain and light respiratory depression, it has attracted much attention since its listing. In previous clinical trials, ciprofol has been shown to have similar safety and tolerability to propofol during induction and maintenance of anaesthesia[9], and ciprofol has potential clinical application value.

Teng Y et al. [10] in the IIa and IIb study of ciprofol concluded that 0.4–0.5 mg/kg ciprofol for colonoscopy had equivalent anesthesia to 2.0 mg/kg propofol and had a similar safety profile with no serious adverse events. This trial investigated the safety of higher doses of ciprofol in gynecologic ambulatory surgery by comparing 0.5 mg/kg ciprofol with 2 mg/kg propofol. In this study, the success rate of induction was 100% in both groups, a result that suggests that ciprofol has good anesthetic efficacy when applied to gynecologic ambulatory surgery. In a phase III clinical trial of gastroscopy [6], the induction time was 1.1 ± 0.5 min in the ciprofol group and 1.1 ± 0.4 min in the propofol group (P = 0.405), and the mean time to complete awakening was 3.3 ± 3.1 min in the ciprofol group compared with 2.0 ± 2.1 min in the propofol group (P < 0.05). In a phase 3 multicenter study of elective surgery[11], the time to successful induction was 0.91 ± 0.03 min in the ciprofol group and 0.80 ± 0.03 min in the propofol group (P < 0.05),and the time to disappearance of the eyelash reflex was 0.80 ± 0.03 min and 0.71 ± 0.03 min(P < 0.05 ). The time of loss of consciousness in this experiment was 1.6 ± 0.4 min in the ciprofol group and 1.4 ± 0.2 min in the propofol group (P < 0.05), which was longer than in the previous study and may be related to the speed of drug injection. In this study, a syringe pump was used to limit the drug infusion to 1 min to reduce the effect of infusion speed on drug onset time.The longer induction time of ciprofol than propofol in this study may be related to the relatively lower lipophilicity of ciprofol due to the introduction of the cyclopropyl structure, which affects the type of formulation (e.g., lower oil content) and reduces the free ciprofol concentration and the rate of ciprofol crossing the blood-brain barrier, etc.

In a study of ciprofol used in gynecological surgery[12], the incidence of adverse events was significantly reduced in the ciprofol group, (20% vs. 48.33%, P = 0.0019), not including injection pain. However, this study included injection pain in the observation of adverse events, so the overall incidence of adverse events was high(56.2% vs. 92.2%,P < 0.05), The incidence of body movement was higher in the ciprofol group than in the propofol group, and the difference was statistically significant. Whether intraoperative body movements are related to our adoption of a shallow depth of anesthesia with a sudden increase in surgical stimulation remains to be observed in further studies.

Injection pain is one of the most common adverse effects in propofol anesthesia.There are many factors influencing injection pain, including injection site and injection speed, venous size, etc. In order to improve patient comfort and reduce patient pain, clinically, anesthesiologists seek different ways to alleviate propofol injection pain, including drug interventions (lidocaine, opioids, dexmedetomidine, propofol medium and long chain fat emulsion injection, etc.), physical interventions (selection of coarser blood vessels, dilution of propofol, low-dose desensitization, etc.),but the effect was not good. In this study, the incidence of ciprofol injection pain was significantly lower than that of the propofol group (1.6% vs. 76.6%), Ciprofol is an isomer of propofol, and the cyclopropyl group is introduced into the chemical structure of propofol, which improves the pharmacological and physicochemical properties, eliciting less pain on injection[7, 10].

Hypotension is also a common adverse effect of propofol. Systolic blood pressure < 90 mmHg is the threshold for associated myocardial and renal injury, and a brief (> 5 min) reduction in systolic blood pressure by 41 ~ 50 mmHg from baseline increases the incidence of myocardial infarction by a factor of 3. Moreover, MAP < 80 mmHg for more than 10 min increases mortality in patients, and the longer the time and the lower the MAP, the greater the risk [13–15], so the inhibition of propofol for circulation is one of the reasons why its use in anesthesia is limited. In this study, the blood pressure in both groups decreased with a similar trend, which mostly decreased within 3 min after drug administration, and then gradually stabilized. Compared with T1, SBP were lower at T2 ~ T9 and the difference between SBP at T 1 and at T 3 ~ T 6 in the ciprofol group was lower than that in the propofol group,which indicates that ciprofol is more beneficial for hemodynamic stability of patients. Adverse events such as hypotension that occurred in both groups recovered on after administration of small amounts of cardiovascular active drugs or within a short period of time without serious adverse consequences.

The present trial had several limitations. First, this study used a cuff for noninvasive blood pressure testing. There is a delay in the observation of blood pressure changes and it is unknown if more severe blood pressure changes have occurred. But performing invasive blood pressure monitoring for short procedures is unnecessarily traumatic for the patient. Second, this trial was conducted in patients with ASA I or II, and further studies are needed in Elderly, frail and seriously ill patients. Third, due to the short duration of the procedure, the use of inotropic drugs may have an impact on the time to awakening.

Overall, the results of this study suggest that ciprofol is as effective as propofol in anesthesia in gynecological ambulatory surgery, while having a lower incidence of adverse events.

## Data Availability

The datasets generated and analysed during the current study are not publicly available due to institutional restrictions but are available from the corresponding author on reasonable request.

## Abbreviations

GABAA: gamma-aminobutyric acid-A
BMI: body mass index
SBP: systolic blood pressure
MAP: mean arterial pressure
DBP: diastolic blood pressure
HR: heart rate
NIBP: non-invasive blood pressure
ECG: electrocardiogram
SpO2: pulse oxygen saturation
BIS: bispectral index
MOAA/S: Modified Observer’s Assessment of Alertness/Sedation
PACU: post anesthesia care unit
HR: heart rate
VT: tidal volume
FiO2: fraction of inspired oxygen
RR: respiratory rate
PETCO2: end-tidal CO2 gas tension
